# The microRNA miR-181c controls microglia-mediated neuronal apoptosis by suppressing tumor necrosis factor

**DOI:** 10.1186/1742-2094-9-211

**Published:** 2012-09-06

**Authors:** Li Zhang, Lian-Yan Dong, Ya-Jian Li, Zhen Hong, Wen-Shi Wei

**Affiliations:** 1 Department of Neurology, Huadong Hospital, Fudan University, 221 West Yan An Road, Shanghai, 200040, China; 2 State Key Laboratory of Medical Neurobiology, Shanghai Medical College and Institutes of Brain Science, Fudan University, 130 Dong An Road, Shanghai, 200032, China; 3 Department of Neurology, Huashan Hospital, Fudan University, 12 Wulumuqi Road Central, Shanghai, 200040, China

**Keywords:** Microglial activation, Hypoxia, Neuronal apoptosis, miR-181c, TNF-α

## Abstract

**Background:**

Post-ischemic microglial activation may contribute to neuronal damage through the release of large amounts of pro-inflammatory cytokines and neurotoxic factors. The involvement of microRNAs (miRNAs) in the pathogenesis of disorders related to the brain and central nervous system has been previously studied, but it remains unknown whether the production of pro-inflammatory cytokines is regulated by miRNAs.

**Methods:**

BV-2 and primary rat microglial cells were activated by exposure to oxygen-glucose deprivation (OGD). Global cerebral ischemia was induced using the four-vessel occlusion (4-VO) model in rats. Induction of pro-inflammatory and neurotoxic factors, such as tumor necrosis factor (TNF)-α, interleukin (IL)-1β, and nitric oxide (NO), were assessed by ELISA, immunofluorescence, and the Griess assay, respectively. The miRNA expression profiles of OGD-activated BV-2 cells were subsequently compared with the profiles of resting cells in a miRNA microarray. BV-2 and primary rat microglial cells were transfected with miR-181c to evaluate its effects on TNF-α production after OGD. In addition, a luciferase reporter assay was conducted to confirm whether TNF-α is a direct target of miR-181c.

**Results:**

OGD induced BV-2 microglial activation *in vitro*, as indicated by the overproduction of TNF-α, IL-1β, and NO. Global cerebral ischemia/reperfusion injury induced microglial activation and the release of pro-inflammatory cytokines in the hippocampus. OGD also downregulated miR-181c expression and upregulated TNF-α expression. Overproduction of TNF-α after OGD-induced microglial activation provoked neuronal apoptosis, whereas the ectopic expression of miR-181c partially protected neurons from cell death caused by OGD-activated microglia. RNAinterference-mediated knockdown of TNF-α phenocopied the effect of miR-181c-mediated neuronal protection, whereas overexpression of TNF-α blocked the miR-181c-dependent suppression of apoptosis. Further studies showed that miR-181c could directly target the 3′-untranslated region of TNF-α mRNA, suppressing its mRNA and protein expression.

**Conclusions:**

Our data suggest a potential role for miR-181c in the regulation of TNF-α expression after ischemia/hypoxia and microglia-mediated neuronal injury.

## Background

Microglia are the resident innate immune cells of the CNS. Microglia display a quiescent phenotype in the healthy brain, but they become highly activated after brain insult, when they produce reactive oxygen and nitrogen species and pro-inflammatory cytokines [1-5]. Therefore, microglial cells play important roles in the surveillance of and response to pathological insults [6]. The available evidence has indicated that microglial activation results in the overproduction of pro-inflammatory cytokines, which may contribute to the development and progression of neurodegenerative disorders [7]. For example, activated microglia and their secreted factors, such as tumor necrosis factor (TNF)-α, are key mediators of neuroinflammation, which have been shown to correlate with neurodegenerative diseases such as Alzheimer’s disease [8,9], Parkinson’s disease [10,11] and HIV-associated dementia [12,13]. In addition, activated microglia, by releasing pro-inflammatory cytokines, participate in the inflammatory response associated with cerebral ischemia [1]. Overproduction of inflammatory factors from ischemia-activated microglia is thought to mediate post-ischemic neuronal damage [14].

MicroRNAs (miRNAs) are a class of small non-coding RNA molecules, 22to 25 nucleotides in length, that function in the post-transcriptional regulation of gene expression. miRNAs bind partly complementary sequences in mRNAs, targeting them for degradation and/or inhibiting their translation, and thereby downregulating the expression of the targeted proteins [15]. Dysregulation of miRNAs has been shown to contribute to many types of human diseases, including neuronaldisorders [16-18]. However, there are almost no data on the miRNA expression profiles of microglial cells exposed to hypoxia. Although microglial activation is considered to be the hallmark of neuroinflammation [7], it remains unknown whether expression of pro-inflammatory cytokines during microglial activation is regulated by miRNAs.

In this study, we used oxygen-glucose deprivation (OGD)-activated microglial cells, which are characterized by the overproduction of pro-inflammatory and neurotoxic factors. Wefound that OGD downregulated miR-181c expression but upregulated TNF-α expression. The overproduction of TNF-α that followed OGD-induced microglial activation induced neuronal apoptosis, whereas ectopic expression of miR-181c partially protected neurons from cell death caused by OGD-activated microglia. Therefore, our data suggest an important role for miR-181c in the regulation of TNF-α expression after ischemia/hypoxia and microglia-mediated neuronal injury.

## Methods

### Reagents

All cell culture products were purchased from Gibco (Carlsbad, CA, USA). The following antibodies were used: anti-CD11b (Millipore, Bedford, MA, USA), anti-TNF-α, anti-inducible nitric oxide synthase (iNOS; Abcam, Cambridge, MA, USA), anti-interleukin (IL)-1β (Santa Cruz Biotechnology, CA, USA), and anti-glyceraldehyde 3-phosphate dehydrogenase (GAPDH; Cell Signaling Technology, Beverly, MA, USA). Recombinant soluble (s)TNF was purchased from Peprotech (Rocky Hill, NJ, USA).

### Microglial cell culture

The Shanghai Medical Experimental Animal Care Committee approved the protocol for this study, and all animal experiments were conducted in accordance with the National Institutes of Health*Guidelines for the Care and Use of Laboratory Animals*.

Primary hippocampal microglial cells were isolated from glial cultures prepared from newborn (less than 24 hours old) Sprague–Dawley (SD) rats(Laboratory Animal Center, Shanghai Medical College, Fudan University, Shanghai, China). Glial cells were cultured in 75 cm^2^ flasks for 14 days in DMEM/F12 (Gibco BRL, Grand Island, NY, USA) supplemented with 10% FCS(Hyclone, Logan, UT, USA), 100 U/ml penicillin and 100 mg/ml streptomycin. Microglia were isolated from primary mixed glial cell cultures on day 10 by shaking the flasks overnight at 300 rpm on a rotary shaker at 37°C. The purity of the microglial cultures was assessed as over 90%, using a CD11b antibody. Cells were cultured for 2 daysbefore treatment.

The BV-2 murine microglial cell line and rat primarycultured microglial cells were maintained in DMEM supplemented with 10% FCS.

### Oxygen-glucose deprivation and preparation of microglial conditioned medium

OGD was performed with the microglial cells as reported previously [19], with minor modifications. Briefly, microglial cells were initially maintainedin serum/glucose-free DMEM in an anoxic (95% N_2_ and 5% CO_2_) environment at 37°C for 1 hour. The cells were then transferred to a normoxic incubator (95% air, 5% CO_2_) and maintained in serum-free defined medium.At 48 hours after OGD, the medium conditioned with the microglial cells was harvested. This fresh, conditioned medium was cleared by centrifugation and diluted 1:1 with serum-free defined medium, then used to treat the neurons For all experiments, conditioned media from the indicated microglia were collected simultaneously and used in parallel.

### Four-vessel occlusion

Transient forebrain ischemia was induced by four-vessel occlusion (4-VO) as described by Pulsinelli [19]. Briefly, male Wistar rats weighing 260 to 320 g were anesthetized with 4% (w/v) chloral hydrate 400 mg/kg administered intraperitoneally. The bilateral vertebral arteries were permanently electrocauterized and the bilateral common carotid arteries (CCAs) were freed,then atraumatic clasps were placed around the CCAs without interrupting the arterial blood flow. The rats were allowed to recover for 24 hours after the surgical incisions were closed. On the next day, the rats were anesthetized, and forebrain ischemia was induced by tightening the clasps for 20 minutes. The clasps were then removed for reperfusion. In sham-operated groups, the same procedure was performed without the 4-VO procedure.

### Tissue preparation

At 3 daysafter ischemia induction, animals were deeply anesthetized, then killed by perfusion with saline, followed by 4% paraformaldehyde in 0.1 mol/L phosphate buffer (pH 7.4). Brains were removed and post-fixed for 4 hours in the same fixative, then embedded in optimum cutting temperature compound, and stored at −70°C. Serial sections (20 μm in thickness) were cut on a cryostat. Only those regions that contained the same coronal plane as the hippocampus were evaluated.

### Immunofluorescence

Sections were incubated with primary antibodies against anti-CD11b (mouse monoclonal antibody; dilution 1:300) and anti-iNOS (rabbit monoclonal; dilution 1:100) overnight at 4°C. After thorough washing, the cells were incubated with fluorescein isothiocyanate-conjugated anti-rabbit IgG (goat, 1:500 dilution; Santa Cruz Biotechnology) or with DyLight 649-conjugated anti-mouse IgG (goat, 1:500 dilution; Santa Cruz Biotechnology) for 1.5 hours at room temperature. Finally, the cells were washed and mounted with mounting medium containing DAPI (Vector Laboratories). A negative control, in which the primary antibody was omitted, was used to test for the specificity of the antibody being used. The images were captured using a conventional fluorescence microscope (Leica, Heerbrugg, Switzerland).

### Primary culturing of hippocampal neurons

Primary hippocampal neuronal cultures were prepared as described previously [20]. Briefly, the hippocampi of newborn (< 24 hours old) SD rats were dissected and incubated in Hank’s balanced salt solution without Ca^2+^ or Mg^2+^. The tissues were subsequently dissociated using 0.125% trypsin (Gibco BRL, Grand Island, NY, USA) by digesting for 15 minutes at 37°C followed by gentle trituration through a flame-polished Pasteur pipette. The dissociated cells were seeded onto glass coverslips coated with poly-L-lysine (molecular weight 30,000 to 70,000, 0.1 mg/ml; Sigma-Aldrich, St. Louis, MO, USA) or into 24-well plates at a density of 1 × 106 cells/ml. The cells were cultured in serum-free medium (Neurobasal-A; Gibco BRL,Gaithersburg, MD, USA) and supplemented with 2% B27 (Gibco BRL), 10 μL/ml penicillin-streptomycin and 2 mmol/l glutamine (Solarbio). The cells were maintained by changing the medium every 2–3 days and incubating at 37.8°C in a humidified atmosphere containing 5% CO_2_. The purity of the neuronal cultures was determined by immunocytochemical staining using an antibody against neurofilament proteins (a neuron-specific marker). and more than 90% of the cells were found to be neurons.

### Hoechst staining assay for apoptosis

For Hoechst 33342 staining, neurons were fixed in 4% paraformaldehyde and incubated for 10 minutes at room temperature with 5 μg/ml Hoechst 33342 (Sigma Chemical Co, St. Louis, MO). Cells were analyzed using a conventional fluorescence microscope (Leica), and the neurons with clearly condensed and segmented chromatin were counted as apoptotic. For all of the groups, 300 cells (including the normal and apoptotic cells) were counted manner using fluorescence microscopy by a researcher blinded to the treatment given. Three independent experiments were performed for each group.

### RNA isolation, microarrays, and real-timePCR

Total RNA was extracted from the BV-2 or primary cultured cells (both with and without OGD treatment) using TRIzol reagent (Invitrogen, San Diego, CA, USA) in accordance with the manufacturer’s instructions. Small RNAs were isolated using a commercial kit (miRNA Isolation Kit; Ambion, Inc., Austin, TX, USA). Array experiments were performed by a company (CapitalBio Corp., Beijing, China) as described on the company’s website ( http://www.capitalbio.com) and in previous reports [21,22], using an miRNA array (GeneChip miRNA Array; Affymetrix Inc., Santa Clara, CA, USA),which is composed of 6,703 probe sets from the miRNAs registered in the Sanger miRBase miRNA database (version 11; http://microrna.sanger.ac.uk; accessed 15 April 15 2008). Semi-quantitative real-time PCR, using SYBR Green I, was conducted to compare the relative expression levels of specific mRNAs, as described previously [23]. *Taq*Man miRNA assays (Applied Biosystems Inc., Carlsbad, CA, USA) were used to quantify mature miRNA expression levels,in accordance with the manufacturer’s protocol. For each of the selected miRNAs, real-time PCR measurements were performed to obtain a mean C_T_ value for each sample. The C_T_ valuesof the different samples were compared using the 2-^ΔΔCT^ method [24], and U6 expression levels were used as an internal reference.

### Western blotting analysis

Western blotting was performed as described previously [25]. Briefly, total protein was extracted from cultured cells and quantified using a commercial bicinchoninic acid (BCA) kit(BCA Protein Assay Kit; Pierce Biotechnology Inc., Rockford, IL, USA) with BSA as the standard. Equal amounts of protein from different cells were separated by 10% SDS-PAGE and transferred to a nitrocellulose membrane (Bio-Rad, Hercules, CA, USA). The membrane was blocked with 5% non-fat milk, and incubated with primary antibodies against TNF-α (rabbit polyclonal; 1:500 dilution) and GAPDH (rabbit monoclonal; 1:3000 dilution). The blots were then incubated for 2 hours at room temperature with horseradish peroxidase-conjugated secondary antibodies (goat; 1:3000 dilution) in blocking buffer and appropriate secondary antibodies. Target proteins were detected using an enhanced chemiluminescence kit (Amersham Pharmacia Biotech, Uppsala, Sweden).

### Measurement of cytokine release by enzyme-linked immunosorbent assay

To detect the levels of pro-inflammatory factors in the hippocampus, rats were killed at defined time points. Brain homogenates were obtained from the hippocampi ,and separated by centrifugation at 14,000 *g* for 5 minutes at 4°C to remove cellular debris. The supernatant was stored at −80°C until use. To detect cytokine secretion in microglial supernatants in response to OGD, cells were plated intosix-well plates and exposed to OGD or normoxic conditions, as described above. After 48 hours, the media were collected and stored at −80°C until use. The concentrations of IL-1β and TNF-α in the stored media were measured using a sandwich ELISA kit (DuoSet; R&D Systems, Minneapolis, MN, USA).

### Nitric oxide production assay

Nitric oxide (NO) levels were determined by measuring the levels of the stable metabolite nitrite in the culture medium, as described previously [26]. Briefly, BV-2 or primary rat microglial cells in 24-well plates (2 × 10^5^ cells in 500 ml/well) were exposed to OGD. Sample aliquots (100 ml) were mixed with 100 ml Griess reagent and incubated at 25°C for 10 minutes. Absorbance at 550 nm was measured on a microplate reader.

### Oligonucleotide transfection

The miRNA duplexes corresponding to miR-181c were designed as described previously [27]. The control RNA duplexes (referred to as NC for ‘negative control’) for the miRNA mimics and the small interfering RNAs (siRNAs) were not homologous to any human gene sequences. The siRNAs were synthesized by targeting mouse TNF-α (GenBank accessnumber NM_013693) transcripts. The sequences of the RNA oligoribonucleotides (Genepharma, Shanghai, China)are shown in Table 1. Oligonucleotide transfection was performed with commercialreagents (Lipofectamine 2000; Invitrogen Corp.). Each transfection used 50 nmol/L of RNA duplexes.

**Table 1:** The sequences of the RNA oligoribonucleotides used for transfection

**Name**	**Direction**	**Sequence (5′→3′)**
miR-181c mimic	Forward	AACAUUCAACCUGUCGGUGAGU
	Reverse	UCACCGACAGGUUGAAUGUUUU
NC	Forward	UUCUCCGAACGUGUCACGUTT
	Reverse	ACGUGACACGUUCGGAGAATT
TNF-α siRNA	Forward	UGAGGUCAAUCUGCCCAAGUACUUA
	Reverse	UAAGUACUUGGGCAGAUUGACCUCA

miR, microRNA; NC, negative control; TNF-α siRNA, tumor necrosis factor-α small interfering RNA.

### Vector construction and luciferase reporter assays

To generate the miR-181c expression vector, the miR-181c gene was amplified from mouse genomic DNA and cloned into the pcDNA3.0 vector (Invitrogen Corp.). The open reading frame of the mouse TNF-α gene was amplified and cloned into the pcDNA3.0 vector. The luciferase complexes were constructed by ligating oligonucleotides containing the wild-type or mutated putative target site of the mouse TNF-α 3′-untranslated region (UTR) into the multi-cloning site of the p-MIR luciferase reporter vector (Ambion Inc., Austin, TX, USA). HEK293 or BV-2 cells were cotransfected with 80 ng of the luciferase reporter plasmid, 40 ng of the pRL-TK-Renilla-luciferase plasmid (Promega Corp., Madison, WI, USA), and the indicated RNAs (final concentration 20 nmol/l). At 24 hours after the transfection, the firefly and Renilla luciferase activities were measured (Dual-Luciferase Reporter Assay; Promega Corp.). Each transfection was repeated twice in triplicate.

### Statistical analyses

All data are expressed as the mean ± SE of at least three independent experiments performed in triplicate. Statistical analyses were performed using ANOVA models and Student’s *t*-tests. *P* < 0.05 were considered significant.

## Results

### Hypoxia/ischemia induces microglial activation and the release of pro-inflammatory cytokines and neurotoxic factors

**Figure 1:**
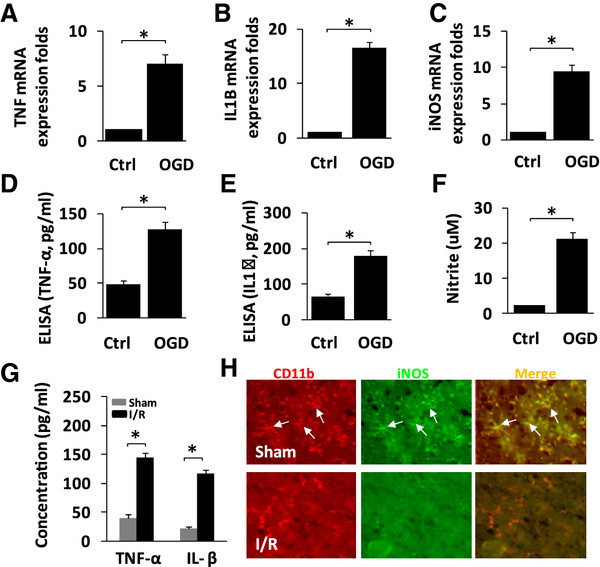
**Hypoxia/ischemia induces microglial activation and the release of pro-inflammatory cytokines and neurotoxic factors.** (**A**-**C**) BV-2 cells were exposed to oxygen-glucose deprivation (OGD), and the mRNA levels of the pro-inflammatory cytokines (**A**)tumor necrosis factor (TNF)-α, (**B**) interleukin (IL)-1β and (**C**)inducible nitric oxide synthase (iNOS) were evaluated using real-time PCR at 3 hoursafter OGD treatment. (**D**-**E**) The release of(**D**) TNF-α (**E**) and IL-1β into the medium of BV-2 cells was measured by ELISA at 48 hours after OGD. (**F**) The release of NO into the medium of BV-2 cells was measured by the Griess assay. Results are presented as the mean ± SE from three independent experiments. (**G**) Brain homogenates were obtained from the hippocampus 3 daysafter transient global cerebral ischemia/reperfusion (I/R) injury. The supernatant concentrations of IL-1β and TNF-α were tested by ELISA. (**H**) Sections from rat brain taken 3 daysafter I/R injury were incubated with primary antibodies against CD11b and iNOS. Representative immunoreactivities in the hippocampal CA1 region are shown. Photomicrographs are shown at ×400 magnification. ‘Ctrl’ (control) represents the microglial cells that were not subjected to OGD treatment. *P < 0.05.

Microglia activated by ischemic conditions have been shown to release pro-inflammatory and neurotoxic factors, such as IL-1, TNF-α and NO, which may be responsible for severe brain-tissue damage [2,3]. Therefore, real-time PCR and ELISA were used to assess the induction of IL-1β and TNF-α in BV-2 microglia exposed to OGD. BV-2 cells were activated after OGD (Figure 1), characterized by the overexpression of IL-1β and TNF-α mRNA as detected by real-time PCR, and by the secretion of the cytokines IL-1β and TNF-α as detected by ELISA. In addition, OGD caused expression of iNOSand mRNA, and overproduction of the neurotoxic factor, NO (Figure 1C,F).

We further confirmed these effects in a rat model. We induced 20 minutes of global cerebral ischemia by 4-VO in rats, and studied the microglial activation and release of pro-inflammatory cytokines in the hippocampus. We evaluated microglial activation 3 days afterI/R. We detected increased levels of IL-1β and TNF-α in the hippocampus by ELISA (Figure 1G). After 3 days of I/R, the number of microglia was markedly increased in the hippocampal CA1 region. By contrast, only a few scattered ramified microglia (resting microglia) were seen in sham-operated rats (Figure 1H). In addition, immunofluorescence showed that transient global cerebral I/R significantly increased expression of iNOS in the hippocampus (Figure 1H). These results indicate that hypoxia/ischemia induces microglial activation and the release of pro-inflammatory cytokines and neurotoxic factors.

### Oxygen-glucose deprivation upregulates tumor necrosis factor-α expression and downregulates microRNA (miR)-181c expression in BV-2 cells

**Figure 2:**
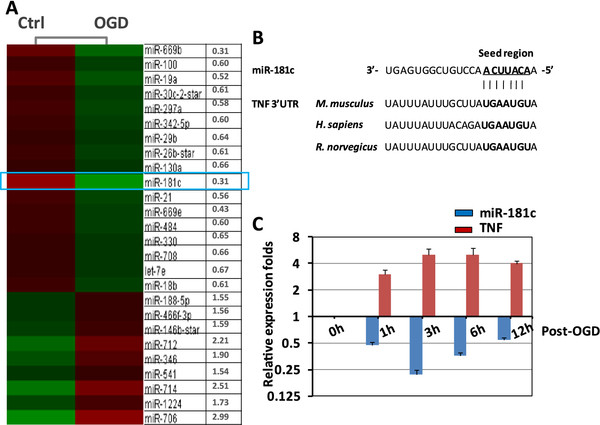
**Oxygen-glucose deprivation (OGD) upregulates tumor necrosis factor (TNF)-α expression and downregulates microRNA (miR)-181c expression in BV-2 cells.** (**A**) BV-2 cells were exposed to OGD for 3 hours. The miRNA was isolated, and the expression profiles of OGD-activated BV-2 cells were compared with those of resting cells. A heat map of the 26 differentially expressed miRNAs (9 upregulated and 17 downregulated) is shown. (**B**) Alignment of the predicted miRNA binding sites in the 3′-UTR of the TNF-α mRNA. (**C**) Relative expression levels of BV-2 TNF-α mRNA and miR-181c at different time points after OGD treatment. Control cells were not subjected to OGD (0 hours). The results are presented as the mean ± SE from three independent experiments. ‘Ctrl’ (control) represents the control microglial cells that were not subjected to OGD treatment.

We further evaluated the miRNA expression profiles of the OGD-activated BV-2 cells compared with the resting cells by miRNA microarray. The thresholdfoldchange value to screen for upregulated and downregulated miRNAs was ≥1.5. In total, 26 miRNAs that exhibited significantly altered expression levels between the hypoxic and normoxic conditions were identified. Of these 26 miRNAs, 7 were upregulated and 19 were downregulated. miR-181c exhibited the highest degree of downregulation in the activated microglia (Figure 2A). Subsequent database searches of both the TargetScan ( http://www.targetscan.org) and Sanger ( http://microrna.sanger.ac.uk) sitesshowed that TNF-α mRNAs have conserved miR-181c recognition sites in their 3′-UTRs (Figure 2B). It is generally accepted that miRNAs exert their function by downregulating the expression of their downstream target genes. To confirm this inverse correlation between the expression of miR-181c and TNF-α found in this study, we measured the mRNA expression levels of TNF-α at different time points after OGD treatment. TNF-α exhibited increased levels of mRNA expression, whereas expression of miR-181c was significantly decreased after treatment (Figure 2C). These results confirmed an inverse correlation between expression of miR-181c and TNF-α during hypoxia-induced microglial activation.

### Tumor necrosis factor-α is a direct target of microRNA (miR)-181c

**Figure 3:**
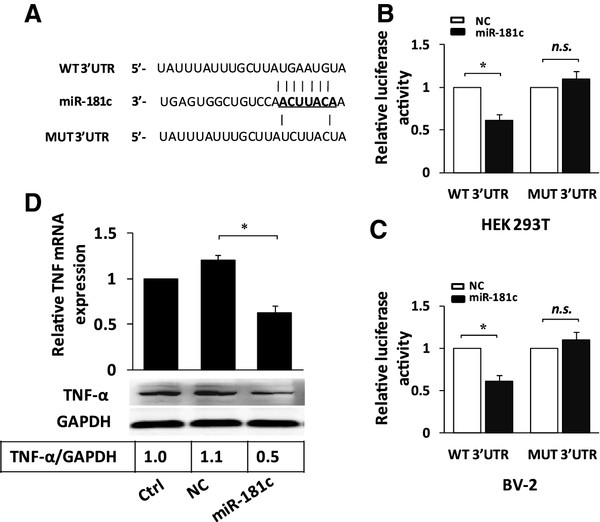
**tumor necrosis factor-α is a direct target of microRNA (miR)-181c.** (**A**) A mouse TNF-α 3′-UTR fragment containing wild-type or mutated miR-181c-binding sites was cloned downstream of the luciferase reporter gene. Mutations were generated in the TNF-α 3′-UTR sequences complementary to the seed region of miR-181c, as indicated. (**B**-**C**) Luciferase activity assays using reporters with wild-type or mutant mouse TNF-α 3′-UTRs were performed after co-transfection with miR-181c mimics or NC in HEK293 (**B**) or BV-2 (**C**) cells. The luciferase activity of the NC transfection in each experiment was used to normalize the data; the luciferase activity of the NC transfection was set to 1. (**D**) BV-2 cells were transfected with miR-181c mimics or NC. After 48 hours, cells were harvested, and the Expression levels of TNF-α mRNA and protein were evaluated by real-time PCR and western blotting. The results are presented as the mean ± SE from three independent experiments. Ctrl represents the control microglial cells that were not subjected to OGD treatment; NC represents the negative control for the miR-181c mimics. *P < 0.05. NS, not significant.

As detailed above, OGD upregulated TNF-α and downregulated miR-181c expression in microglial cells. We hypothesized that increased TNF-α production might result from the downregulation of miR-181c. To address this hypothesis, a dual-luciferase reporter system was used (Figure 3A). Coexpression of miR-181c significantly suppressed the firefly luciferase reporter activity of the wild-type 3′-UTR but not of the mutant 3′-UTR in both HEK193T (Figure 3B) and BV-2 cells (Figure 3C), indicating that miR-181c suppresses TNF-α expression through miRNA binding sequences in its 3′-UTR. Finally, BV-2 cells were transfected with the NC or miR-181c duplexes. miR-181c was capable of significantly suppressing the mRNA and protein expression of TNF-α (Figure 3D). Taken together, this result suggests that miR-181c suppresses TNF-α expression by binding to the 3′-UTR, and that TNF-α is a direct target of miR-181c.

### Ectopic expression of microRNA (miR)-181c attenuates oxygen-glucose deprivation-activated BV2-induced neuronal apoptosis

**Figure 4:**
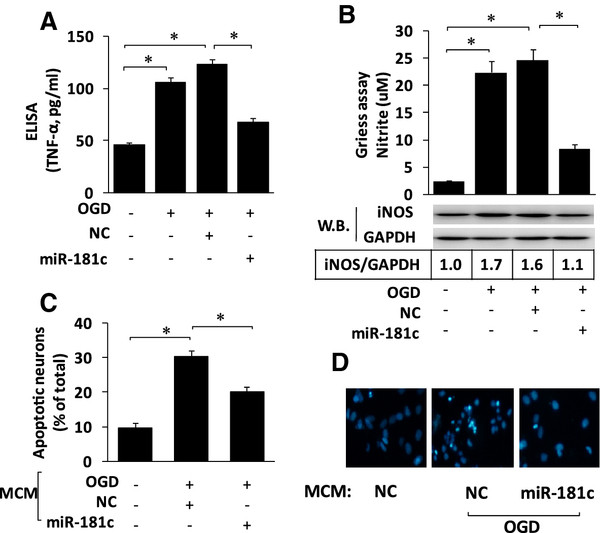
**Ectopic expression of microRNA (miR)-181c attenuates oxygen-glucose deprivation-activated BV2-induced neuronal apoptosis.** (**A**-**B**) BV-2 cells were transfected with miR-181c mimics or NC 24 hoursbefore activation by OGD. After 48 hours, the production levels of TNF-α (**A**) and NO (**B**, top) were determined by ELISA and the Griess assay, respectively. The cells were harvested at 48 hours after OGD, and iNOS protein expression was evaluated by western blotting (**B**, lower). (**C**-**D**) BV-2 cells were transfected with miR-181c mimics or NC 24 hoursbefore activation by OGD. After 48 hours, the conditioned media were harvested. Cultured neurons were grown in the microglia-conditioned medium (MCM) and then analyzed for apoptosis using Hoechst 33342 staining. The percentage of apoptotic cells in the total neuronal population was calculated (**C**). Representative photographs are shown in (**D**). The results are presented as the mean ± SE from three independent experiments. NC represents thenegative control for the miR-181c mimics. *P < 0.05.

TNF-α is a key pro-inflammatory cytokine, and increased levels of this cytokine have been associated with the pathology of a variety of neurological, neurodegenerative, and neurotoxic conditions [28]. TNF-α can activate receptor-mediated pro-apoptotic pathways within the neuron. TNF-α can also stimulate microglial activation in the form of iNOS induction, which leads to the production of NO, a neurotoxic factor associated with brain injury [29]. Therefore, we transfected microglial cells with NC or miR-181c mimics and treated the microglial cells with OGD. Evaluation of the harvested microglia-conditioned mediumshowed that the miR-181c-transfected cells secreted less TNF-α after OGD (Figure 4A). In addition, the miR-181c-transfected cells exhibited decreased iNOS expression and NO production (Figure 4B). To confirm whether the ectopic miR-181c expression was able to attenuate activated microglia-induced neuronal death, neurons were exposed to cell-free conditioned medium collected from microglia with or without the ectopic expression of miR-181c. The ectopic expression of miR-181c significantly reduced the neuronal apoptosis induced by OGD-activated microglia (Figure 4C,D), suggesting that ectopic expression of miR-181c attenuates OGD-activated BV2-induced neuronal apoptosis.

### microRNA (miR)-181c controls microglia-mediated neuronal apoptosis and is dependent on tumor necrosis factor-α

**Figure 5:**
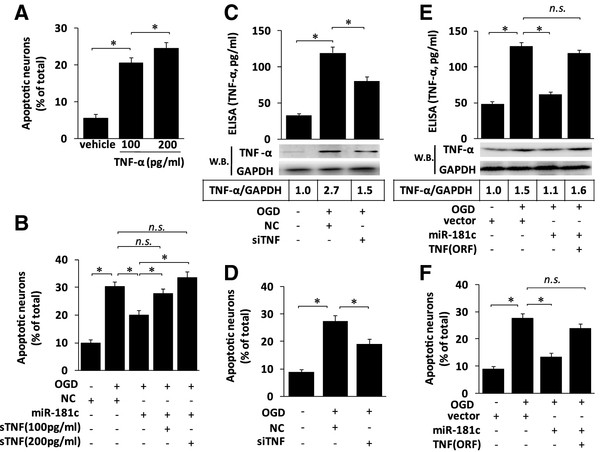
**microRNA (miR)-181c controls microglia-mediated neuronal apoptosis dependent on tumor necrosis factor (TNF)-α.** (**A**) Neurons were treated with vehicle or soluble (s)TNF-α as indicated. After 48 hours, the percentage of apoptotic cells in the total neuronal population was calculated. (**B**) BV-2 cells were transfected with negative control (NC) or miR-181c as indicated before being subjected to OGD. After 48 hours, the conditioned medium (CM) was harvested. sTNF-α was added to the CM at the indicated concentration before cultured neurons were grown in microglia-conditioned medium (MCM) for 48 hours, and neuronal apoptosis was detected using Hoechst 33342 staining. (**C**-**D**) BV-2 cells were transfected with NC or small interfering TNF before they were subjected to OGD. (**C**) TNF-α protein expression levels were determined by western blotting after 24 hours, and TNF-α release levels were determined by ELISA after 48 hours. (**D**) Cultured neurons were then grown in the MCM for 48 hours, and neuronal apoptosis was detected using Hoechst 33342 staining. (**E**, **F**) BV-2 cells were transfected with NC, miR-181c or TNF-α (open reading frame without the 3′-untranslated region) expression vectors as indicated, before being subjected to OGD. (**E**) TNF-α protein expression levels were determined by western blotting after 24 hours, and TNF-α release levels were determined by ELISA after 48 hours. (**F**) Cultured neurons were then grown in MCM for 48 hours, and neuronal apoptosis was detected using Hoechst 33342 staining. The results are presented as the mean ± SE from three independent experiments.NC represents the negative control for the miR-181c mimics. *P < 0.05. NS, not significant.

To clarify the role of microglia-derived TNF-α in miR-181c-mediated neuronal apoptosis, we first tested the capacity of TNF-α to trigger death in cultured neurons. We found that addition of soluble (s)TNF-α to the neuron culture triggered apoptosis in a dose-dependent manner (Figure 5A). Furthermore, addition of sTNF-α significantly abrogated miR-181c-mediated neuronal survival (Figure 5B). Next, the effects of TNF-α silencing on neuronal apoptosis were determined. We found that silencing of TNF-α in the microglia-conditioned media significantly decreased both TNF-α production (Figure 5C) and microglia-mediated neuronal apoptosis (Figure 5D), which was similar to the phenotype induced by miR-181c. By contrast, the ectopic expression of TNF-α using a TNF-α expression vector that encoded the entire coding sequence of TNF but lacked its 3′-UTR expression significantly abrogated miR-181c-induced neuronal survival (Figure 5E,F), indicating that TNF-α is a functional target for miR-181c.

### microRNA (miR)-181c controls tumor necrosis factor-α production in primary rat microglial cells

**Figure 6:**
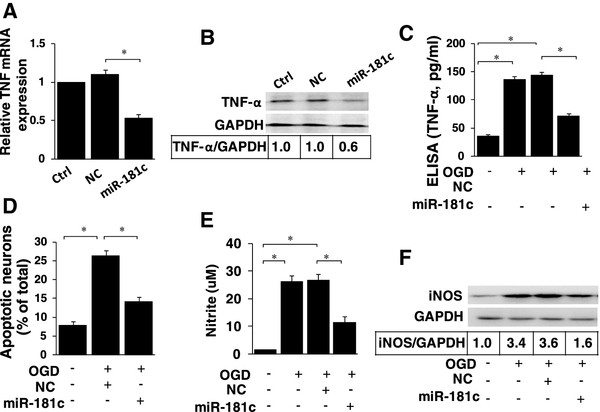
**microRNA (miR)-181c controls tumor necrosis factor-α production in primary rat microglial cells.** (**A**-**B**) Primary rat microglia cells were transfected with miR-181c mimics or NC. After 48 hours, cells were harvested, and the TNF-α mRNA (A) and protein (**B**) expression levels were evaluated by real-time PCR and western blotting. (**C**-**D**) Primary rat microglial cells were transfected with miR-181c mimics or NC 24 hoursbefore activation by OGD. After 48 hours, the production of TNF-α was determined by ELISA (**C**). Cultured neurons were then grown in the microglia-conditioned medium (MCM) for 48 hours, and neuronal apoptosis was detected using Hoechst 33342 staining (**D**). (**E**-**F**) Primary rat microglia cells were transfected with miR-181c mimics or NC 24 hoursbefore activation by OGD. After 48 hours, the production of NO was determined by the Griess assay (**E**). The cells were harvested, and iNOS protein expression was evaluated by western blotting (**F**). The results are presented as the mean ± SE from three independent experiments. Ctrl represents the microglial cells that were not treated with oligonucleotide transfection; NC represents the negative control for the miR-181c mimics. *P < 0.05. NS, not significant.

**Figure 7:**
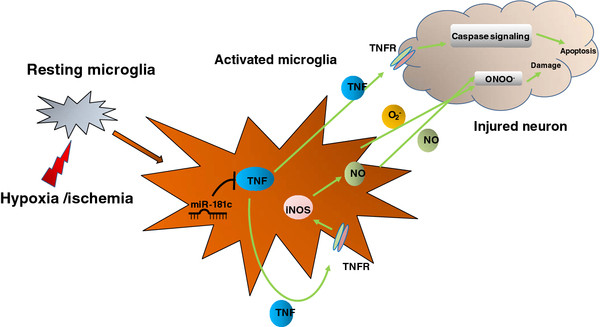
**A schematic of the role for microRNA (miR)-181c-tumor necrosis factor (TNF)-α in microglia-mediated neuronal injury.** Hypoxia-ischemia resulted in microglia activation and miR-181c downregulation. Downregulation of miR-181c leads to increased TNF-α production, as TNF-α is a direct target of miR-181c. TNF-α can activate receptor-mediated proapoptotic pathways within the neuron. TNF-α can also stimulate microglia activation in the form of inducible nitric oxide synthase (iNOS) induction, which leads to production of nitric oxide (NO) Finally, the interaction between NO and superoxide anion (O2^−^) forms toxic peroxynitrite (ONOO^−^), which also causes neuron damage.

To confirm the observations from the primary rat microglia, primary-culturenewborn rat microglial cells were used. As seenwith the BV-2 cells, miR-181c transfection significantly suppressed basal TNF-α mRNA and protein expression levels (Figure 6A,B). In addition, OGD led to overproduction of secreted TNF-α in the primary rat microglia, whereas ectopic expression of miR-181c significantly reduced TNF-α production (Figure 6C). This system was also used to confirm that ectopic expression of miR-181c-attenuated neuronal death was caused by activated primary rat microglia (Figure 6D). Finally, as seen with the BV-2 cells, we found that miR-181c inhibited OGD-induced iNOS expression and NO production in primary rat microglia (Figure 6E,F). Taken together, the results indicate that miR-181c controls microglia-mediated neuronal apoptosis by suppressing TNF-α production (Figure 7).

## Discussion

To our knowledge, this study is the first to report the miRNA expression profile of microglial cells exposed to ischemia, although it is already becoming clear that miRNA-dependent post-transcriptional gene regulation plays pivotal roles at all stages of neural development [30]. In addition, for the first time, we have also identified in microglia a microRNA, miR-181c, whichcan directly regulate TNF-α production post-transcription.

Inflammation is an underlying component of a diverse range of neurodegenerative diseases and their associated neuropathologies. Increasing evidence suggests that microglia are a key causative factor in this process [7,31]. Activated microglia serve initially to phagocytose necrotic debris, which allows for the eventual healing of the injured brain. However, under pathological conditions, activated microglia can induce significant and strongly detrimental neurotoxic effects through excess production of a large array of cytotoxic factors, such as IL-1β, NO, reactive oxygen species (ROS), and TNF-α [1,32]. In our study, we found that OGD upregulated IL-1β, TNF-α, and iNOS. However, although microglial activation is considered to be a hallmark of neuroinflammation, whether the expression of pro-inflammatory cytokines is regulated by miRNAs during microglial activation has not been previously determined. Therefore, after identifying the miRNA profile of ischemia-activated microglia, we investigated the correlation between altered miRNA expression and pro-inflammatory cytokines.

TNF-α is a key pro-inflammatory cytokine that has been reported to play an important role in the events that follow ischemia [33]. Increased levels of TNF-α have been associated with the pathological effects of a variety of infectious, neurological, neurodegenerative, and neurotoxic conditions [33]. We analyzed the predicted TNF-α-regulating miRNAs that were downregulated in activated microglial cells, and found that TNF-α might be regulated by miR-181c. To better understand this regulatory relationship, the capacity of miR-181c to directly regulate TNF-α expression by binding to its 3′-UTR was confirmed. To determine whether the dysregulated miRNAs were functional, we confirmed that ectopic expression of miR-181c resulted in decreased release of TNF-α from the microglial cells and decreased neuronal apoptosis. Silencing TNF-α also produced a significant decrease in neuronal apoptosis in microglia-conditioned media, which was similar to the phenotype induced by miR-181c. Additionally, ectopic TNF-α expression significantly abrogated the neuronal survivalinducec by miR-181c. All these results suggest that miRNAs represent endogenous functional microglial moleculesthat respond to ischemia and produce a neurotoxic phenotype in microglia. Taken together, the results from our study suggest that some miRNAs lie upstream of pro-inflammatory cytokines or other inflammatory mediators. However, we cannot exclude the possibility that some of the miRNAs that were upregulated in the activated microglia play a role because they may act upstream of several anti-inflammatory genes.

An increasing body of evidence indicates that oxidative stress resulting from excessive generation of ROS and reactive nitrogen species (RNS) is a causal factor in various neurodegenerative disorders [34]. The interaction between NO and superoxide anion-forming toxic peroxynitritehas been proposed as being involved in neuronal injury [35,36]. TNF-α can activate receptor-mediated proapoptotic pathways within the neuron, and can further stimulate the microglia through iNOS and cyclooxygenase 2 induction [28,29]. Therefore, inhibition of TNF-α production is expected to be beneficial in inflammation-related neurological disorders. In our study, we found that OGD resulted in increased production of NO, whereas ectopic expression of miR-181c could suppress expression of iNOS, leading to decreased production of NO. Therefore, our study indicates an important role of miR-181c in TNF-α-mediated neurotoxicity after ischemia (Figure 7).

Our study also demonstrates the importance of investigating the mechanisms underlying cytokine regulation when studying the effect of cytokines on neuronal injury, because this information may facilitate the development of more effective therapeutic interventions. Previous studies have shown enhancement of neuronal damage by TNF-α *in vivo* in ischemia models. For example, intracerebroventricular administration of TNF-α increased the lesional area produced by focal ischemia, whereas inhibition of TNF-α activity by administration of soluble TNF-α receptors or anti-TNF-α antibodies reduced the ischemic damage [37]. Clinical studies on the effects produced by modification of cytokines in CNS disease are currently underway. At present, the main challenge is to deliver sufficient levels of cytokines or their modifiers to the ischemic or injured brain. miRNAs may represent a new drug target class. By taking advantage of their small size and the current knowledge of miRNA biogenesis, modified RNAs can be transiently delivered as synthetic, pre-processed miRNAs or as anti-miRNA oligonucleotides [38].

Delivering an miRNA that reduces the protein levels of target genes linked to a particular disease by post-transcriptional regulation or that targets a particular functional miRNA linked to a particular disease represents new therapeutic options. A recent study has shown that the *in vivo* administration of miR-124 suppresses experimental autoimmune encephalitisby affecting macrophages, suggesting that miRNA delivery could be used to treat some inflammatory diseases associated with microglial activation [39]. A recent report also showed that another member of the miR-181c family, miR-181a, could influence cerebral ischemia outcomes *in vitro* and *in vivo* by regulating GRP78 expression in astrocytes [40]. Collectively, all of these studies have indicated that manipulating miRNA levels could be a potential treatment strategy for ischemic brain injury.

## Conclusions

We identified a correlation between miRNA levels and the expression of genes involved in pro-inflammatory cytokine production. We also characterized one particular molecular series, the miR-181c-TNF-α pathway, which is partially responsible for microglia-mediated neuronal apoptosis. Our data suggest a potential role for miR-181c in the regulation of TNF-α expression after ischemia/hypoxia and microglia-mediated neuronal injury.

## Abbreviations

3′-UTR: 3′-untranslated region; BSA: bovine serum albumin; CM: conditioned media; CNS: central nervous system; DAPI: 4',6-diamidino-2-phenylindole; ECL: enhanced chemiluminescence; ELISA: enzyme-linked immunosorbent assay; FCS: fetal calf serum; IL-1β: interleukin-1β; iNOS: inducible nitric oxide synthase; MCM: microglia-conditioned medium; miRNA: microRNA; NC: negative control; NO: nitric oxide; OGD: oxygen-glucose deprivation; RNS: reactive nitrogen species; ROS: reactive oxygen species; siRNA: small interfering RNA; TNF: tumor necrosis factor.

## Competing interests

The authors declare no competing financial interests.

## Authors’ contributions

LZ and WSW designed the research; LZ, LYD, YJL, ZH, and WSW performed the research; LZ, LYD, YJL, and WSW analyzed data; and LZ and WSW wrote the paper. All authors read and approved the final manuscript.
